# Lysophosphatidic acid as a regulator of endometrial connective tissue growth factor and prostaglandin secretion during estrous cycle and endometrosis in the mare

**DOI:** 10.1186/s12917-020-02562-6

**Published:** 2020-09-17

**Authors:** Anna Szóstek-Mioduchowska, Natalia Leciejewska, Beata Zelmańska, Joanna Staszkiewicz-Chodor, Graça Ferreira-Dias, Dariusz Skarzynski

**Affiliations:** 1 Department of Reproductive Immunology and Pathology, Institute of Animal Reproduction and Food Research of Polish Academy of Sciences, Olsztyn, Tuwima-st 10, 10-748 Olsztyn, Poland; 2 Department of Animal Physiology and Biochemistry and Biostructure, Faculty of Veterinary Medicine and Animal Science, Poznan University of Life Sciences, Poznan, Poland; 3 CIISA, Centre for Interdisciplinary Research in Animal Health, Faculty of Veterinary Medicine, University of Lisbon, Lisbon, Portugal

**Keywords:** LPA, CTGF, Prostaglandin, Mare, Endometrosis

## Abstract

**Background:**

Equine endometrosis is a chronic degenerative condition, described as endometrial fibrosis that forms in the stroma, under the basement membrane and around the endometrial glands. The role of lysophosphatidic acid (LPA) in the development of tissue fibrosis varies depending on the organ, and its profibrotic role in mare endometrosis remains unclear. The study aimed to establish the endometrial presence of LPA and its receptors (LPAR1–4), together with its effects on connective tissue growth factor (CTGF) and prostaglandins (PG) secretion from equine endometrium under physiological (estrous cycle), or pathological conditions (endometrosis). Mare endometria in the mid-luteal phase (*n* = 5 for each category I, IIA, IIB, III of Kenney and Doig) and in the follicular phase (n = 5 for each category I, IIA, III and *n* = 4 for IIB) were used. In experiment 1, the levels of LPA, *LPAR1–4* mRNA level and protein abundance were investigated in endometria at different stages of endometrosis. In experiment 2, the in vitro effect of LPA (10^− 9^ M) on the secretion of CTGF and PGs from endometrial tissue explants at different stages of endometrosis were determined.

**Results:**

Endometrial LPA concentration was higher in the mid-luteal phase compared to the follicular phase in category I endometrium (*P* < 0.01). There was an alteration in endometrial concentrations of LPA and LPAR1–4 protein abundance in the follicular phase at different stages of endometrosis (*P* < 0.05). Additionally, LPA increased the secretion of PGE_2_ from category I endometrium in both phases of the estrous cycle (*P* < 0.05). The effect of LPA on the secretion of CTGF and PGF_2α_ from endometrial tissue was altered depending on different stages of endometrosis (*P* < 0.05).

**Conclusion:**

Our data indicate that endometrosis disturbs proper endometrial function and is associated with altered endometrial LPA concentration, its receptor expression and protein abundance, PGE_2_/PGF_2α_ ratio, and CTGF secretion in response to LPA. These changes could influence several physiological events occurring in endometrium in mare during estrous cycle and early pregnancy.

## Background

Lysophosphatidic acid (LPA) is a small phospholipid present in many mammalian cells and tissues [1]. Lysophosphatidic acid affects cell migration, survival, proliferation, changes in the cytoskeleton and also cellular interactions, which are crucial for many physiological processes [2]. The biological activity of LPA is mediated by activation of its six receptors, LPAR1 to LPAR6, which mediate diverse biological actions. The broad expression of LPARs in tissues and their coupling to a number of types of G proteins (Gq, Gi, Gs, G12/13) underlies their wide variety of cellular and biological activities [1, 3]. Lysophosphatidic acid receptors 1 to 3 are structurally related and included to the endothelial differentiation gene family (EDG). Lysophosphatidic acid receptor 1 is extensively expressed, with strong expression in the brain, colon, heart, placenta, small intestine and prostate [4]. Lysophosphatidic acid receptors 2 and 3 display a more restricted pattern of expression and are expressed in the testis, kidney, heart, lung and brain [5, 6]. High levels of *LPAR4* mRNA are expressed in the mammalian ovary [7]. Physiologically, LPA and its active LPARs were found in female reproductive organs, such as the uterus [8–11], ovary [12, 13], oviduct [14] and placenta [15] in human, mouse, pig, sheep and cow. Studies have indicated significant importance of receptor-mediated LPA signaling in mammalian reproduction [8–12]. In cattle, pigs and sheep, LPA is a meaningful mediatorin the control of uterine functions during the estrous cycle and early pregnancy in particular implantation via its effect on endometrial prostaglandin (PG) secretion [9, 16, 17]. Prostaglandins play important role in the proliferation of cells, angiogenesis in the endometrium, implantation of embryos and regulation of the lifespan of the corpus luteum (CL) [18–21].

Besides the above-mentioned role of LPA under physiological condition, LPA was shown to be an important player in the development of tissue fibrosis [22–24]. The importance of LPA in the development of fibrosis varies depending on the organ but its profibrotic effect remains unclear and requires better understanding [22–24]. In vitro studies have shown that in the lungs and peritoneum, LPA may contribute to fibrosis by its effect on connective tissue growth factor (CTGF) secretion and promoting fibroblast proliferation [25, 26]. The high abundance of CTGF was confirmed in the uterine flushing proteome of mares with fibrotic endometrial degeneration and with endometritis [27]. Additionally, LPA induces mesangial cell proliferation, their contractility and PGE_2_ synthesis [28, 29]. To the best of our knowledge, the endometrial concentration of LPA and its receptor expression, as well as the effects of LPA on CTGF and PG at different stages of equine endometrosis has not yet been described. Equine endometrosis is a chronic degenerative condition, described as fibrosis that forms in the endometrial stroma, under the basement membrane and around the endometrial glands, frequently connected with pathological alteration of glands within fibrotic foci [30–32]. Endometrosis affects secretory function of endometrial cells, causing alteration in the uterine microenvironment and dysfunction in processes occurring in early pregnancy [32–35]. Endometrosis is one of the main reasons for subfertility/infertility in mares and causes a significant economic loss to the horse-breeding industry. In our study, we hypothesized that (1) LPA can affect PG and CTGF secretion from mare endometrium during the estrous cycle; and (2) endometrial level of LPA changes at different stages of endometrosis and enhances CTGF and PGF_2α_ secretion from endometrial tissue. Thus, the study was aimed to establish the endometrial presence of LPA and its receptors (LPAR1–4), together with its effects on PGs and CTGF secretion from equine endometrium under physiological (estrous cycle), or pathological conditions (endometrosis).

## Results

### Experiment 1. Endometrial LPA concentration, LPAR1–4 mRNA levels and protein abundance at different stages of mare endometrosis

#### LPA

**Fig. 1:**
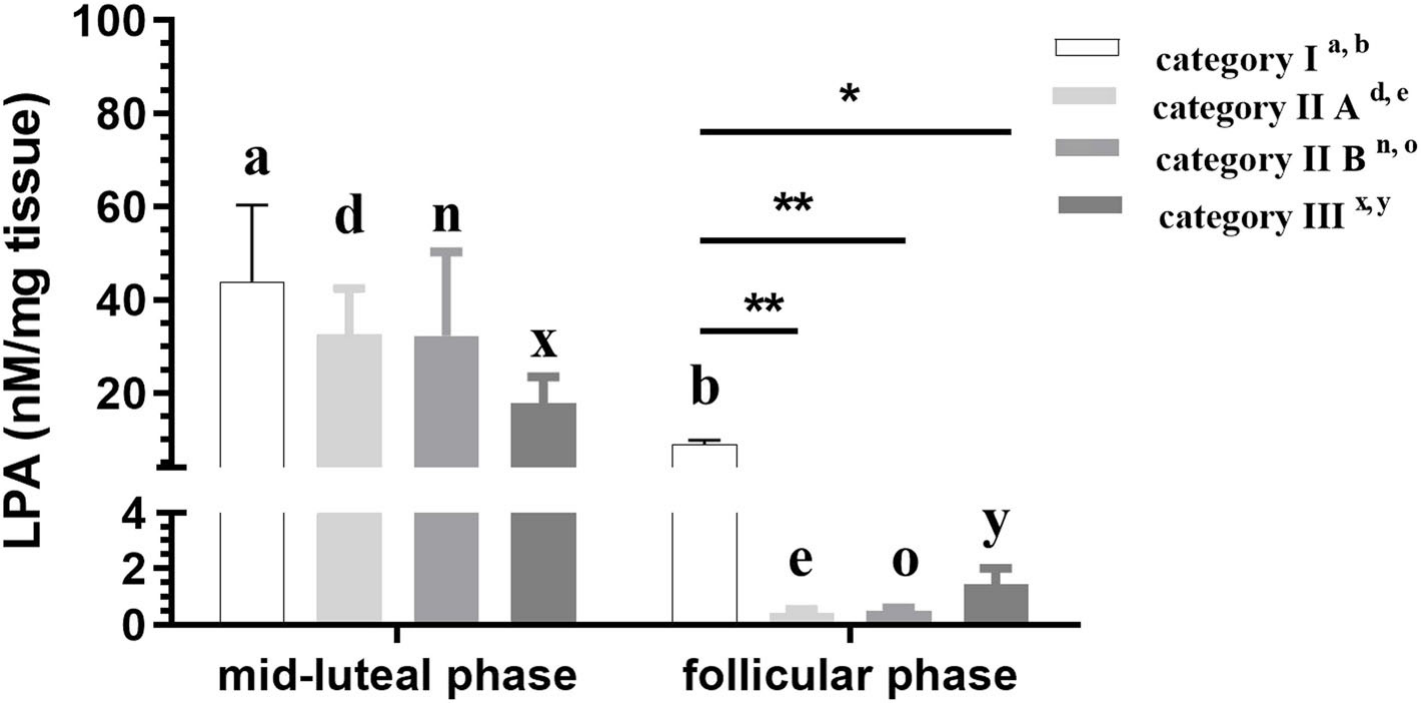
Endometrial LPA level during the mid-luteal and follicular phase at different stages of endometrosis (Kenney and Doig’s endometrium categories I, IIA and IIB and III) in equine endometrium. Superscript letters indicate statistical differences between the mid-luteal and follicular phase in Kenney and Doig’s category I ^a,b^ category IIA ^d,e^ category IIB ^n,o^ and category III ^x,y^. Asterisks indicate statistical differences between LPA levels in endometrosis, within the mid-luteal or follicular phase (*P < 0.05; **P < 0.01)

In categories I, IIA, IIB and III endometria, LPA levels were lower in the follicular phase compared to the mid-luteal phase of the estrous cycle (*P* < 0.01; Fig. 1). In the follicular phase, the level of LPA was lower in categories IIA, IIB and III endometria compared to category I endometrium (P < 0.01, P < 0.01, *P* < 0.05, respectively; Fig. 1).

#### LPAR1

**Fig. 2:**
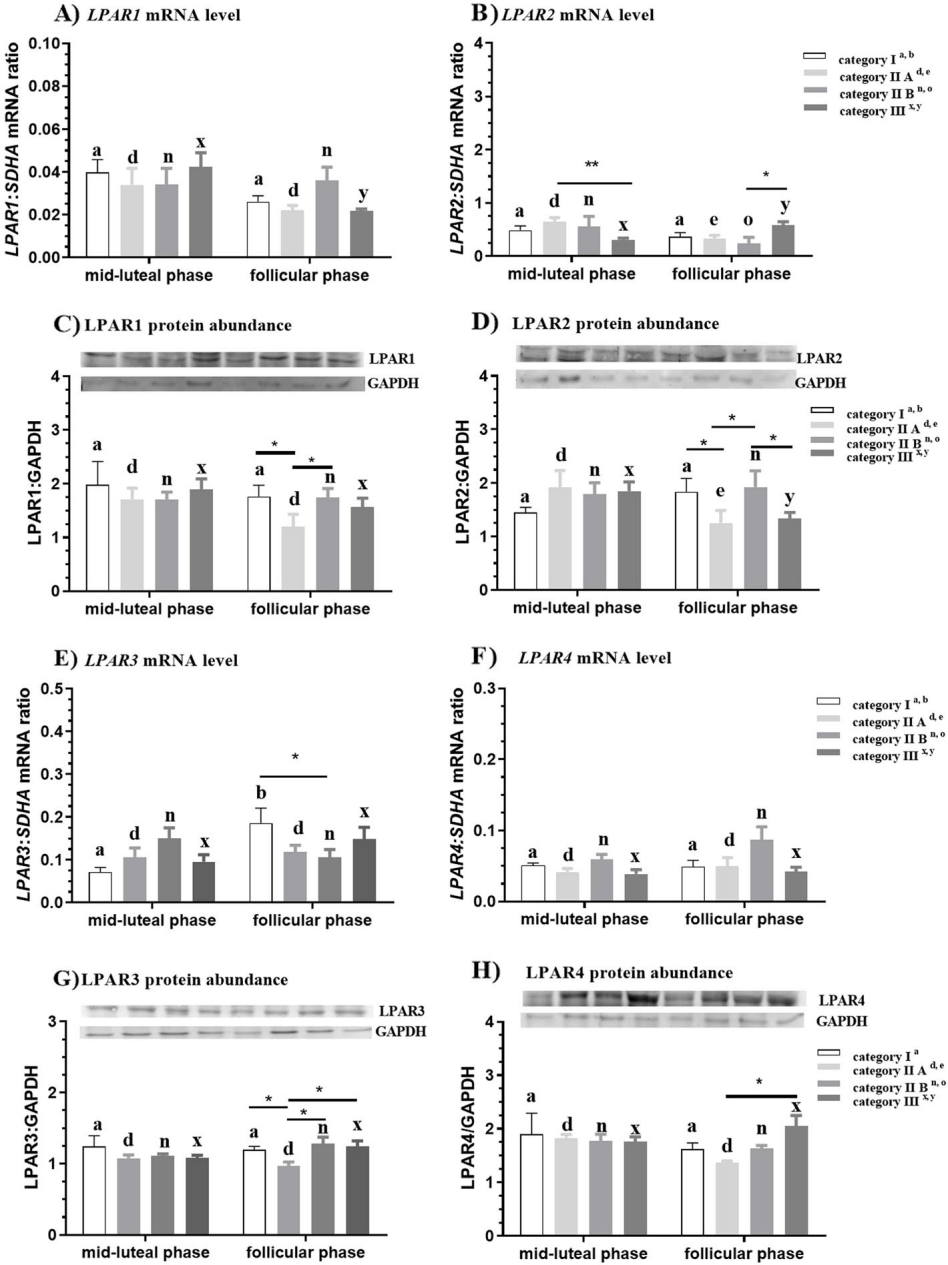
*LPAR1, LPAR2, LPAR3, LPAR4* mRNA level (**a**, **b**, **e**, **f**) and protein abundance (**c**, **d**, **g**, **h**) during the mid-luteal and follicular phase at different stages of endometrosis (Kenney and Doig’s endometrium categories I, IIA and IIB and III) in equine endometrium. Superscript letters indicate statistical differences between the mid-luteal and follicular phase in Kenney and Doig’s category I ^a,b^ category IIA ^d,e^ category IIB ^n,o^ and category III ^x, y^. Asterisks indicate statistical differences between LPAR1, LPAR2, LPAR3, LPAR4 mRNA level/protein abundance in endometrosis, within the mid-luteal or follicular phase (*P < 0.05; **P < 0.01)

In category III endometrium, *LPAR1* mRNA level was higher in the mid-luteal phase compared to the follicular phase (*P* < 0.05; Fig. 2a). The LPAR1 protein abundance was lower in category IIA endometrium compared to category I and IIB endometria in the follicular phase (*P* < 0.05; Fig. 2c).

#### LPAR2

In category IIA and IIB endometria, *LPAR2* mRNA level was higher in the mid-luteal phase compared to the follicular phase (P < 0.05; P < 0.05; Fig. 2b). In category III endometrium, *LPAR2* mRNA level was lower in the mid-luteal phase compared to the follicular phase (*P* < 0.01; Fig. 2b). Additionally, in the mid-luteal phase, *LPAR2* mRNA level decreased in category III, with respect to category IIA endometrium (P < 0.01; Fig. 2b). In the follicular phase*, LPAR2* mRNA level was lower in category IIB in comparison to category III endometrium (*P* < 0.05; Fig. 2b).

In category IIA and III endometria, LPAR2 protein abundance was higher in the mid-luteal phase compared to the follicular phase (*P* < 0.05; P < 0.05; Fig. 2d). In the follicular phase*,* LPAR2 protein abundance was lower in category IIA and III endometria compared to category I (*P* < 0.05; P < 0.05; Fig. 2d). Additionally, in the follicular phase, LPAR2 protein abundance was lower in category III compared to category IIB (*P* < 0.05; Fig. 2d).

#### LPAR3

In category I endometrium, *LPAR3* mRNA level was lower in the mid-luteal phase compared to the follicular phase (*P* < 0.05; Fig. 2e). In the follicular phase, *LPAR3* mRNA level was higher in category I compared to category III endometrium (P < 0.05; Fig. 2e). The *LPAR3* protein abundance was lower in category IIA endometrium compared to categories I, IIB and III endometria in the follicular phase (*P* < 0.05; Fig. 2g).

#### LPAR4

In the follicular phase, LPAR4 protein abundance was lower in category IIA endometrium compared to category III endometrium (P < 0.05; Fig. 2h).

### Experiment 2. The effect of LPA on CTGF and PG secretion at different stages of mare endometrosis

The basal secretion of PGE_2_, PGF_2α_ and CTGF in mid-luteal and follicular phase of the estrous cycle at different stages of endometrosis was shown in Table 1. The basal secretion of PGE_2_ was higher in categories IIA (Table 1; *P* < 0.05), IIB (Table 1; P < 0.05) and III (Table 1; *P* < 0.0001) than in category I in the mid-luteal phase, or in category III compared to category I endometrium in the follicular phase of the estrous cycle (Table 1; *P* < 0.01). The basal secretion of PGF_2α_ increased in category IIB compared to category I in the mid-luteal phase of the estrous cycle (Table 1; P < 0.01), and in category III endometrium with respect to category I endometrium in the follicular phase of the estrous cycle (Table 1.; *P* < 0.05).

**Table 1:** Basal secretion (mean ± standard deviation) of prostaglandin (PG)E_2_, PGF_2α_ and connective tissue growth factor (CTGF) from equine endometrium in mid-luteal phase and follicular phase of the estrous cycle at different stages of endometrosis. Superscript letters indicate significant differences between different stages of endometrosis within each phase of estrous cycle

	mid-luteal phase of estrous cycle			
Category according to Kenney and Doig	I	II A	II B	III
PGE_2_ (ng/g tissue)	408.54 ± 42.04^a^	1116 ± 405.1^b^	1201 ± 111.5^b^	2090 ± 211.4^c^
PGF_2α_ (ng/g tissue)	1590 ± 222.1^a^	1895 ± 311.4^a^	2907 ± 567.4^b^	1865 ± 401.1^a^
CTGF (ng/g tissue)	8.148 ± 3.27^a^	6.563 ± 0.864^a^	6.076 ± 4.007^a^	6.123 ± 1.511^a^
	follicular phase of estrous cycle			
PGE_2_ (ng/g tissue)	1460 ± 494.9^a^	2374 ± 385.3^ab^	1657 ± 365.1^a^	4296 ± 2307^b^
PGF_2α_ (ng/g tissue)	1310 ± 225.2^a^	1586 ± 285.6^ab^	2245 ± 636.8^ab^	3237 ± 1793^b^
CTGF (ng/g tissue)	5.717 ± 3.534^a^	4.510 ± 1.300^a^	7.399 ± 3.692^a^	4.710 ± 1.001^a^

**Fig. 3:**
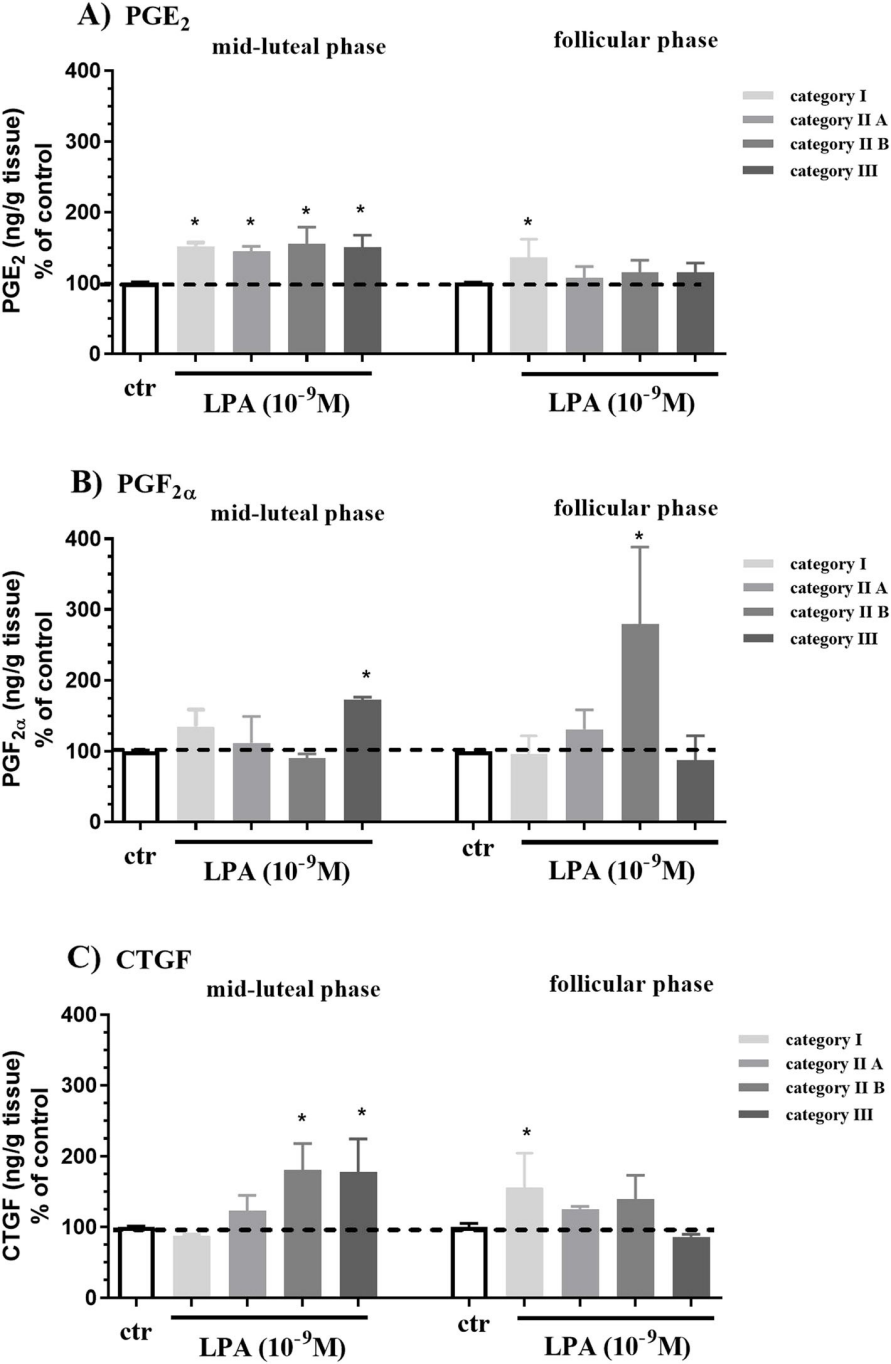
Effect of LPA (10^− 9^ M) on (**a**) PGE_2_, (**b**) PGF_2α_, (**c**) CTGF secretion by endometrial explants from mid-luteal and follicular phase of the estrous cycle at different stages of endometrosis. The endometrial explants were assigned to categories I, IIA, IIB and III according to Kenney and Doig. All values are expressed as % of control. The effect of LPA on PG and CTGF secretion in comparison to control group within each category was analyzed by nonparametric Mann-Whitney U test. Asterisks indicate statistical differences within each of Kenney and Doig category (*P < 0.05)

Lysophosphatidic acid stimulated PGE_2_ secretion from endometrial tissue in all equine endometrial categories, in comparison to the respective control group in the mid-luteal phase (Fig. 3a; *P* < 0.05; category I – 152.1%; II A – 144.8%; II B- 155.6%; II – 150.3% of respective control). Additionally, LPA stimulated PGE_2_ secretion from endometrial tissue in category I compared to the respective control group in the follicular phase (Fig. 3a; *P* < 0.05; 136.6% of respective control).

Lysophosphatidic acid increased PGF_2α_ secretion from endometrial tissue in category III in the mid-luteal phase (Fig. 3b; *P* < 0.05; 172.89% of respective control) and from category IIB in the follicular phase (Fig. 3b; P < 0.05; 279.1% of respective control) compared to the respective control groups. Lysophosphatidic acid increased CTGF secretion from endometrial tissue in category IIB and III in the mid-luteal phase (Fig. 3c; *P* < 0.05; category II B – 180.7%; category III − 177.6% of respective control) and in category I in the follicular phase, compared to the respective control groups (Fig. 3c; *P* < 0.05; 155.5%).

**Fig. 4:**
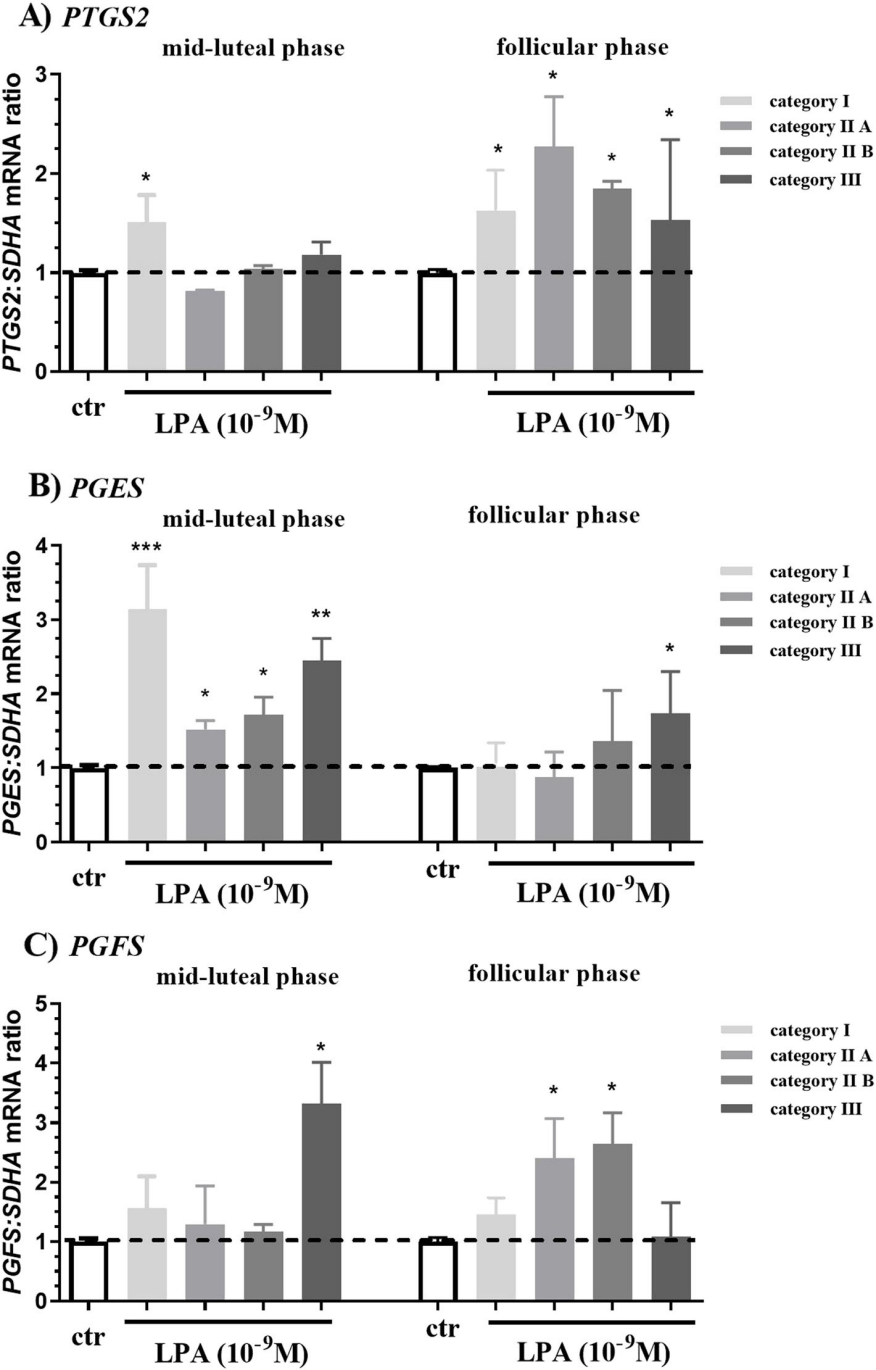
Effect of LPA (10^− 9^ M) on **a**) *PTGS2*, **b**) *PGES*, **c**) *PGFS* mRNA level in endometrial tissue in the mid-luteal and follicular phase of the estrous cycle at different stages of endometrosis. The endometria were assigned to categories I, IIA, IIB and III according to Kenney and Doig. All values are expressed as a fold change. The effect of LPA on *PG synthases* mRNA level in comparison to control group within each category was analyzed by nonparametric Mann-Whitney U test. Asterisks indicate statistical differences between the groups (*P < 0.05)

Lysophosphatidic acid up-regulated *prostaglandin-endoperoxide synthase 2* (*PTGS2)* mRNA level in endometrial tissue in category I (1.5-fold change) in mid-luteal phase (Fig. 4a; *P* < 0.05) and in categories I (1.6-fold change), IIA (2.3- fold change), IIB (1.8-fold change) and III (1.6-fold change) in the follicular phase (Fig. 4a; P < 0.05) compared to the respective control group. Additionally, LPA up-regulated *prostaglandin E*_*2*_ *synthase* (*PGES)* mRNA level in endometrial tissue in categories I (3.14-fold change), IIA (1.5-fold change), IIB (1.7-fold change) and III (2.4-fold change) in the mid-luteal phase, and also in category III (1.7-fold change) endometrium in the follicular phase relative to the respective control group (Fig. 4b; P < 0.05).

Lysophosphatidic acid up-regulated *prostaglandin F*_*2α*_ *synthase* (*PGFS)* mRNA level in endometrial tissue in category III (3.31-fold change), in comparison to their respective control groups in the mid-luteal phase (Fig. 4c; P < 0.05). Additionally, LPA up-regulated *PGFS* mRNA level in endometrial tissue in categories IIA (2.4-fold change) and IIB (2.6-fold change) in the follicular phase compared to the respective control groups (Fig. 4c; P < 0.05).

## Discussion

The role of LPA in mare reproduction remains unclear. To the best of our knowledge, our study is the first one to examine the level of LPA, LPAR1–4 mRNA level and protein abundance as well as their action in endometrium in the mid-luteal and follicular phase of the estrous cycle in mare. In the present study, we showed that endometrial concentration of LPA is higher in the mid-luteal phase compared to the follicular phase, indicating its potential role during this period of estrous cycle. We also demonstrated that in category I endometrium LPA increased PGE_2_ secretion in both follicular and mid-luteal phases, and CTGF only in the follicular phase of the estrous cycle.

The cyclic changes and proper functioning of endometrium depend on the concentration of hormones and their balance. A change in the expression of any of the players might impact on the expression of other factors, which might threaten endometrial homeostasis and many physiological processes. The data obtained in this study indicate that the PGE_2_/PGF_2α_ ratio and CTGF secretion in response to LPA are disturbed at different stages of endometrosis, which may affect the microenvironment of mare endometrium. Additionally, the protein abundance of LPR1–4 is reduced mainly in category IIA endometria in the follicular phase of the estrous cycle. Generally, LPA plays a key role in embryonic development, implantation, and pregnancy establishment in many species [8, 17, 36]. In porcine endometrium, LPA up-regulated PTGS2 expression, which suggests an important role in endometrial function and conceptus development in this species [9]. In turn, in bovine endometrium, LPA stimulated PGE_2_ synthesis and inhibited in vitro PGF_2α_ synthesis on days 16–18 of the estrous cycle and pregnancy [36]. Additionally, LPA has been shown to be an important regulator of cell proliferation [37], angiogenesis [38], inflammation [39], tissue repair and remodeling [40, 41] in different tissues suggesting its involvement in these processes in mare endometrium. Moreover, this phospholipid programs fibroblasts to produce a plethora of paracrine mediators of tissue remodeling, angiogenesis, inflammation, such as chemokines, growth factors, cytokines, pro-angiogenic and pro-fibrotic factors, components of the plasminogen activator system and metallopeptidases (MMP) [41]. These processes and paracrine mediators are essential modulators of endometrium function during the estrous cycle, as well as in early pregnancy. Thus, the alteration of LPA concentration and action at the different stages of endometrosis may disturb these physiological processes.

In the present study, we have shown that CTGF is secreted in response to LPA in the follicular phase in category I endometrium, in contrast to other stages of endometrosis. The physiological role of CTGF in mare endometrium in not yet understood. The pattern of CTGF expression by porcine endometrial cells suggests the involvement of this factor in stroma remodeling and angiogenesis [42]. In the mare, CTGF seems to play an important role in the non-pregnant uterus, while its expression is decreased during early pregnancy [43–45]. Since in mare healthy endometrium LPA stimulated CTGF production, it might be suggested that this cytokine is an important regulator of endometrial cell proliferation, remodeling and angiogenesis, as described for other species [42–45]. In addition, our findings suggest that these processes can be disturbed by changes in CTGF secretion in response to LPA at different stages of endometrosis.

The profibrotic effect of LPA and the augmented expression of LPA and LPARs have been demonstrated in the arterial, lung and liver fibrosis in studies in human and animal models [22, 23, 46–48]. But, our study showed that while in the mid-luteal phase the endometrial level of LPA was not changed at different stages of endometrosis, in the follicular phase, the level of LPA was lower at different stages of endometrosis, in comparison to category I endometrium. Although the level of LPA did not increase at different stages of fibrosis, it is possible that LPA indirectly stimulates fibrogenesis by acting on PGF_2α_ and CTGF. The present study showed that LPA increased CTGF in vitro secretion from category IIB and III endometria in the mid-luteal phase. It also raised PGF_2α_ secretion from category III endometrium in the mid-luteal phase and category IIB endometrium in the follicular phase of the estrous cycle. These players were reported as important factors in the development of fibrosis [49–58]. Prostaglandin F_2α_ has been demonstrated as an inductor of fibrogenesis in several pathogenic conditions, such as pulmonary and myocardial fibrosis and is often linked to disease severity and prognosis [49–51]. In the mare, PGF_2α_ has been shown to increase the expression of collagen 1 and MMPs in endometrial fibroblast [52]. Likewise, CTGF supports glomerular and renal interstitial fibrosis in different kidney diseases [53]. Other studies showed that LPA might contribute to fibrosis by stimulation of CTGF, driving fibroblast proliferation in a paracrine manner [26]. This cytokine affects cell proliferation, migration, and differentiation and promotes the progression of fibrosis, either directly [54], or indirectly, by acting as a downstream regulator of TGF-β1 [55, 56]. Additionally, CTGF up-regulated fibrosis markers (TGF-β1 and tissue matrix metalloproteinase inhibitor-1), collagen *(COL)1* and *COL3* mRNA levels, and decreased PGE_2_ production from mare endometrial explants [57, 58].

In our experimental design, we examined endometrial samples from mares with different degree of fibrosis. Since endometria were obtained *post-mortem*, it was not feasible to evaluate the evolution of the fibrogenic process in individual mares. The current design may have affected the results, and thus it could explain the inconsistent pattern of our observed changes. Possibly, our inconsistent results could be also ascribed to cellular adaptation to achieve a new steady state compatible with cell viability in the new environment. Therefore, further studies should be carried out to elucidate our findings.

## Conclusion

In category I endometrium, LPA concentration increased in the mid-luteal phase compared to the follicular phase of the estrous cycle and LPA increased CTGF and PGE_2_ secretion. Additionally, concentrations of endometrial LPA and LPAR1–4 mRNA level and protein abundance in the follicular phase were altered at different stages of endometrosis. The effect of LPA on the secretion of CTGF and PG from endometrial tissue was changed at different stages of endometrosis. Our data indicate that endometrial degeneration disturbs proper endometrial function and thus changed endometrial LPA concentration, its receptor expression and protein abundance, PGE_2_/PGF_2α_ ratio and CTGF secretion in response to LPA treatment. These changes could influence several physiological events occurring in endometrium in mare during estrous cycle and early pregnancy such as cell proliferation, angiogenesis, inflammation, tissue repair and tissue remodeling.

## Methods

### Endometrial material

The endometrial tissues (*n* = 39; Table 2) were retrieved *post-mortem* from the uterus of cyclic mares (Polish cold-blood horse) at different ages (2–15) weighing around 500–700 kg, at a commercial abattoir euthanized according to European Legislation (EFSA, AHAW/04–027) with protection of animal welfare in order to eliminate pain and suffering of animals. All procedures for the collection of material were accepted by the Local Ethics Committee for Experiments on Animals in Olsztyn, Poland (Agreements No. 51/2011). To ensure that a sufficient number of animals would be available for each experimental group around 160 endometrial samples were obtained over reproductive season (April to July). The mares were healthy according to official government veterinary inspection. The mares were raised in a farm as food animals and slaughtered in order to obtain meat. The endometrial samples were obtained average within 5 min of the animals’ slaughter. Just before slaughter, peripheral blood were taken into heparinized tubes for subsequent progesterone (P_4_) analysis. In the present study, the following phases of the estrous cycle were considered: mid-luteal phase (*n* = 5 for each category I, IIA, IIB, III) and follicular phase (n = 5 for each category I, IIA, III and *n* = 4 for category IIB). The same endometria were used in Experiment 1, as well as in Experiment 2. The determination of phase of estrous cycle based on analysis of P_4_ as well as macroscopic observation of the ovaries [59]. At the mid-luteal phase, we confirmed the presence of a well-developed corpus luteum (CL) associated with follicles 15–20 mm in diameter and P4 > 6 ng/mL. The mare ovary in follicular phase we confirmed the absence of an active CL and the presence of a follicle > 35 mm in diameter, with a P4 concentration < 1 ng/mL [59].

**Table 2:** Experimental procedures used for determination of endometrial LPA, LPAR1–4 mRNA level and protein abundance, effect of LPA on PG and CTGF secretion at different stages of endometrosis. Ctr-control, LPA-lysophosphatidic acid, AA- arachidonic acid

	Experiment 1																							
phase of estrous cycle	MID-LUTEAL												FOLLICULAR											
category	I			II A			II B			III			I			II A			II B			III		
sample (n)	n = 5			n = 5			n = 5			n = 5			n = 5			n = 5			n = 4			n = 5		
	Experiment 2																							
phase of estrous cycle	MID-LUTEAL												FOLLICULAR											
category	I			II A			II B			III			I			II A			II B			III		
treatment	c	LPA	AA	c	LPA	AA	c	LPA	AA	c	LPA	AA	c	LPA	AA	c	LPA	AA	c	LPA	AA	c	LPA	AA
sample (n)	5	5	5	5	5	5	5	5	5	5	5	5	5	5	5	5	5	5	4	4	4	5	5	5

Endometrium was resected from the uterine horns, ipsilateral to the CL (mid-luteal phase) or to the growing follicle (follicular phase), washed with cold sterile RNAse-free saline solution, placed into: (i) 4% buffered paraformaldehyde for hematoxylin-eosin staining [59]; (ii) RNAlater (#AM7021; Invitrogen) for *LPAR* mRNA level determination using qPCR; or (iii) liquid nitrogen for LPA concentration measurement and LPAR protein abundance using Western-blot.

For tissue culture, endometrial explants were put into sterile, incomplete (Ca^2+^- and Mg^2+^-free) Hanks’ balanced salt solution (HBSS; H1387; Sigma-Aldrich) containing gentamicin (20 μg/mL; G1272; Sigma-Aldrich) and 0.1% bovine serum albumin (BSA; A9418; Sigma-Aldrich), kept on ice and transported quickly to the laboratory. Additionally, before running any of the experiments, all endometria were confirmed to be free from bacterial endometritis, by microscopic evaluation of endometrial smears collected with sterile swabs before the endometrium pieces were excised from the myometrium and stained with Diff-Quick [60]. The presence of bacteria detected by cytological examination was the criterion for tissue rejection prior to experiments. As previously defined, the presence of more than two neutrophils per four microscopic fields (mag = 400X) indicate an acute endometritis [61].

The in vitro treatment of endometrial explants with LPA was performed before the histopathological categorization of the endometrium according to Kenney and Doig [31]. Endometrosis was graded according to Kenney and Doig’s classification [31], based on the intensity of fibrosis as well as endometrial gland characteristics (glandular nests, glandular hypertrophy, glandular atrophy and glandular cysts), as category I, IIA, IIB or III, corresponding to minimum, mild, moderate or severe lesions of endometrosis, respectively.

Therefore, sufficient number of repetitions of each experiment were carried out using the collected samples (Table 2). All the explants were collected after treatments and were stored at -80 °C until Kenney and Doig category were assessed and the sufficient number of samples were assigned to each group before further analysis. During the experimental work regarding ELISA, radioimmunoassay (RIA) and qPCR, the researchers were blinded to mare endometrial categories of the samples.

### Experimental procedures

#### Experiment 1. Endometrial LPA concentration, LPAR1–4 mRNA levels and protein abundance at different stages of mare endometrosis

Mares from the mid-luteal phase (*n* = 5 for each category I, IIA, IIB, III) and from the follicular phase (n = 5 for each category I, IIA, III and *n* = 4 for category IIB) were used. The determination of endometrial LPA level was performed using ELISA. The concentration of LPA was assessed per 1 mg of tissue. The *LPAR1*–*4* mRNA levels and protein abundance analyses were performed using qPCR and Western-blot, respectively.

#### Experiment 2. The effect of LPA on CTGF and PG secretion at different stages of mare endometrosis

Uteri from mid-luteal phase (n = 5 for each category I, IIA, IIB, III) and follicular phase (n = 5 for each category I, IIA, III and n = 4 for each category IIB) were used. The endometrial tissue was cut into small pieces (~ 50 mg), then rinsed three times in PBS containing gentamicin (20 μg/mL), and placed into culture tubes with 1 mL Dulbecco’s Modified Eagle’s medium without phenol red (Sigma–Aldrich; 2960) supplemented with 0.1% BSA and antibiotic/antimycotic solution. Tissue pieces were preincubated on a shaker inside a tissue culture incubator at 38.0 °C with 5% CO_2_ in air for 1 h. Afterwards, the medium was replaced with fresh DMEM supplemented with 0.1% BSA and antibiotics/antimycotic for further incubation with vehicle (negative control), LPA agonist (1-oleoyl-sn-glycerol 3-phosphate sodium salt; 10^− 9^ M; 857130P; Avanti Polar Lipids) or arachidonic acid (AA; 50 ng/ml; Sigma-Aldrich; SML1395) for 24 h. Each treatment was carried out in triplicate for all samples from each mare. The dose of LPA was chosen in a preliminary study. The responsiveness of tissues was tested by their incubation for 24 h with AA (50 ng/mL) as a positive control. Measurement of PGE_2_ was performed (supplementary data 1). The conditioned culture medium was collected in tubes with 5% ethylenediaminetetraacetic acid (EDTA) and 1% acetylsalicylic acid solution (pH = 3). Samples were kept frozen at − 20 °C until CTGF and PG concentrations were determination using ELISA. The endometrial explants were placed in RNA*later*® (Sigma-Aldrich; R0901), and stored at − 80 °C until determination of *PTGS2*, *PGES* and *PGFS* mRNA levels using qPCR. In order to normalize the results, PG and CTGF concentration was determined per 1 g of tissue.

Viability of endometrial tissues was assessed using AlamarBlue™ (Invitrogen; #DAL1025) in accordance with the manufacturer’s instructions. After a 24 h of incubation of endometrium, its viability was determined and compared to pre-treatment viability within the control and treatment groups.

### Analytic methods

#### LPA extraction

The extraction of LPA from the equine endometrium was performed using a method described previously [62]. Briefly, 1-oleoyl-LPA contained in 100 mg of tissue was extracted with one volume of 1-butanol. After agitation and centrifugation (5 min. at 3000 g), the upper 1-butanol phase was collected and then evaporated under nitrogen at 50 °C.

#### Total RNA extraction and cDNA synthesis

Total RNA was extracted using TRI Reagent® (T9424; Sigma-Aldrich) following the manufacturer’s information. Concentration and quality of RNA were determined spectrophotometrically and by agarose gel electrophoresis. The ratio of absorbance at 260 and 280 nm (A260/280) was approximately 2. One microgram of total RNA was reverse transcribed into cDNA using a QuantiTect Rev. Transcription Kit (205,313; Qiagen) following the manufacturer’s instructions. The cDNA was stored at − 20 °C until qPCR was carried out.

#### qPCR

qPCR was conducted according to Szóstek-Mioduchowska et al. [63]. The primers for *LPAR1–4* were designed using Primer-BLAST and were synthesized by Sigma-Aldrich. All samples were run in duplicates. Table 3 includes primer sequences, length of expected qPCR product and GenBank accession numbers of *LPAR1*, *LPAR2*, *LPAR3*, *LPAR4* and succinate dehydrogenase complex flavoprotein subunit A (*SDHA*; reference gene, chosen as reported previously [34]). Primers for *PTGS2*, *PGES* and *PGFS* were reported previously [34]. qPCR was performed in 384-well plates with an ABI Prism 7900 sequence detection system using SYBR Green PCR master mix (Applied Biosystems, Foster City, CA). The volume of total reaction was 10 μL: 3 μL of cDNA (1 ng), 1 μL of each forward and reverse primers (500 nM) and 5 μL of SYBR Green PCR master mix. Conditions of qPCR reactions were as follows: initial denaturation (2 min at 50 °C; 10 min at 95 °C), followed by 42 cycles of denaturation (15 s at 95 °C) and annealing (1 min at 60 °C). After each qPCR reaction, melting curves were obtained by stepwise increases in temperature from 60 °C to 95 °C to ensure single-product amplification. Nuclease-free water (129,114; Qiagen), instead of cDNA, was used as a negative control. The specificity of the product was also confirmed by electrophoresis on 2% agarose gel.

**Table 3:** Primers used for qPCR for determination of endometrial LPAR1–4 mRNA level at different stages of endometrosis

Gene name	Primer sequence (5′ – 3′)	Amplicon length (bp)	Accession no. GenBank
*SDHA*	GAGGAATGGTCTGGAATACTG	91	DQ402987.1
	GCCTCTGCTCCATAAATCG		
*LPAR1*	TTCAACTCTGCCATGAACC	85	XM_014735958
	GCTGGCAGCAGAGGATCT		
*LPAR2*	CTCAGCCGCTCCTACCTG	71	XM_023625558
	TAGACAGCCACCATGAGCAG		
*LPAR3*	TCGCAGCAGTGATCAAAAAC	122	XM_001917815
	TGGGCCAGTGTTAAACATCA		
*LPAR4*	GGCAATGCTACTGCCAATAA	104	XM_001501421
	TTGGTTATCAGACCCAGGATG		

Data were analyzed using the method described by Zhao and Fernald [64]. The relative concentration of mRNA (R0) for each target and reference gene (*SDHA*) was calculated using the eq. R0 = 1/(1 + E)^Ct^_,_ where, E is the average gene efficiency and Ct is the cycle number at threshold. The relative gene expression was calculated as R0_target gene_/R0_reference_ gene and was expressed in arbitrary units.

#### Western-blot

Western-blot was done as described recently [57]. All samples were run in duplicate. The membranes were collected after electrophoresis and then incubated overnight at 4 °C with LPA_1_ Polyclonal antibody (Cayman; 10,005,280; 1:100), EDG-4 antibody (H-55) (Santa Cruz; sc-25,490; 1:100), LPA_3_ Polyclonal Antibody (Cayman; 10,004,840; 1:100), P2Y9 Antibody (N-20) (Santa Cruz sc-46,021 1:700) and anti-GAPDH antibody [6C5] (Abcam; ab8245; 1:1000). The LPAR1, LPAR2 and LPAR3 proteins were detected by incubating the membranes with secondary monoclonal anti-rabbit alkaline phosphatase-conjugated antibody (Bethyl; A120-212P; 1:20,000) for 1.5 h at room temperature. The protein LPAR4 and GAPDH protein were detected with Goat IgG-heavy and light chain Antibody (Bethyl; A50-100P; 1:50,000) and mouse IgG-heavy and light chain Antibody (Bethyl; A90-116P; 1:5000), respectively, for 1.5 h at room temperature. The immune complexes were visualized using chemiluminescence (SuperSignal™ West Dura Extended Duration Substrate; 34,076; Thermo Scientific). Antibody dilution buffers, instead of the primary antibody solution, were used as a negative control. The secondary antibodies were incubated with the samples according to the above described protocol. This allowed to eliminate non-specific binding or false positives signals due to non-specific binding of the secondary antibody. The blots were scanned, and the specific bands were quantified using the *VersaDoc* MP 4000 System for densitometric analyses. Glyceraldehyde-3-phosphate dehydrogenase (GAPDH) was used as an internal control for protein loading. The original, full-length blots are included in Supplementary data 2.

#### ELISA assay

The concentration of LPA in endometrial tissue was determined using the Lysophosphatidic Acid Assay Kit II (Echelon Biosciences Inc.; #K-2800S) following the manufacturer’s instructions. All samples were run in duplicate. The standard curve for LPA ranged from 0.064 μM to 200 μM. The intra- and inter-assay coefficients of variation (CVs) were 7.3 and 9.2%, respectively.

The concentration of PGE_2_ in the conditioned medium was determined using the Prostaglandin E_2_ EIA kit (Cayman Chemical Company; #514010) following the manufacturer’s instructions. The standard curve for PGE_2_ ranged from 16.5 pg/mL to 1000 pg/mL. The intra- and inter-assay CVs were 8 and 12%, respectively. The concentration of PGF_2α_ in the conditioned medium was determined using the PGF_2α_ ELISA kit (Enzo Life Sciences; ADI-900-069) following the manufacturer’s instruction. The standard curve for PGF_2α_ ranged from 3 pg/mL to 50,000 pg/mL. The intra- and inter-assay CVs were 9 and 11%, respectively. The concentration of CTGF in the conditioned medium was determined using the Enzyme-linked Immunosorbent Assay Kit for Connective Tissue Growth Factor (Cloud-Clone; SEA010Po) following the manufacturer’s instruction. The standard curve for CTGF ranged from 0.63 ng/mL to 40 ng/mL. The intra- and inter-assay CVs were 8 and 11%, respectively.

#### Radioimmunoassay

Plasma concentrations of P_4_ were assayed by RIA (Diasource; KIP1458). All samples were run in duplicate. The P_4_ standard curve ranged from 0.12 to 36 ng/mL. The intra- and inter-assay CVs were on average 8 and 10%, respectively.

#### Statistical analysis

For each statistical analysis, a Gaussian distribution was tested using the D’Agostino & Pearson normality test (GraphPad Software version 7; GraphPad, San Diego, CA, USA). The data are shown as mean ± standard deviation (SD). The data were not transformed. Data from Experiment 2 are expressed as fold change. In Experiment 1. a two-way ANOVA followed by Bonferroni multiple comparison test was used. Differences in Kenney and Doig’s category I, II A, IIB and III endometria within mid-luteal and follicular phase of the estrous cycle, and between those groups were assessed. In Experiment 2, the effect of LPA on gene expression and PG and CTGF secretion in comparison to the control group within each category was analyzed by the nonparametric Mann-Whitney U test. The basal secretion of PGE_2_, PGF_2α_ and CTGF between different stages of endometrosis, within each phase of estrous cycle, was analyzed using one-way ANOVA followed by Tukey’s multiple comparisons test. The results were considered significantly different at *P* < 0.05.

## Supplementary information

**Additional file 1.**

**Additional file 2.**

## Data Availability

The data that support the findings of this study are available from the corresponding author upon reasonable request.

## Abbreviations

BSA: Bovine serum albumin
CL: Corpus luteum
COL: Collagen
CTGF: Connective tissue growth factor
GAPDH: Glyceraldehyde-3-phosphate dehydrogenase
LPA: Lysophosphatidic acid
PG: Prostaglandin
PTGS2: Prostaglandin-endoperoxide synthase 2
PGES: Prostaglandin E_2_ synthase
PGFS: Prostaglandin F_2α_ synthase
SDHA: Succinate dehydrogenase complex flavoprotein subunit A
