# Supplementary material for: Lysophosphatidic acid as a regulator of endometrial connective tissue growth factor and prostaglandin secretion during estrous cycle and endometrosis in the mare
Source: BMC Vet Res. 2020 Sep 17;16:343. doi: 10.1186/s12917-020-02562-6 (PMC7499873; doi:10.1186/s12917-020-02562-6)

This additional file includes original, full-length blot images presented in Figure 2.

In each gel, the order of samples is the same as in the paper: Category I, IIA, IIB, III (mid-luteal phase) and Category I, IIA, IIB, III (follicular phase of estrous cycle).

GAPDH Fig. 2C

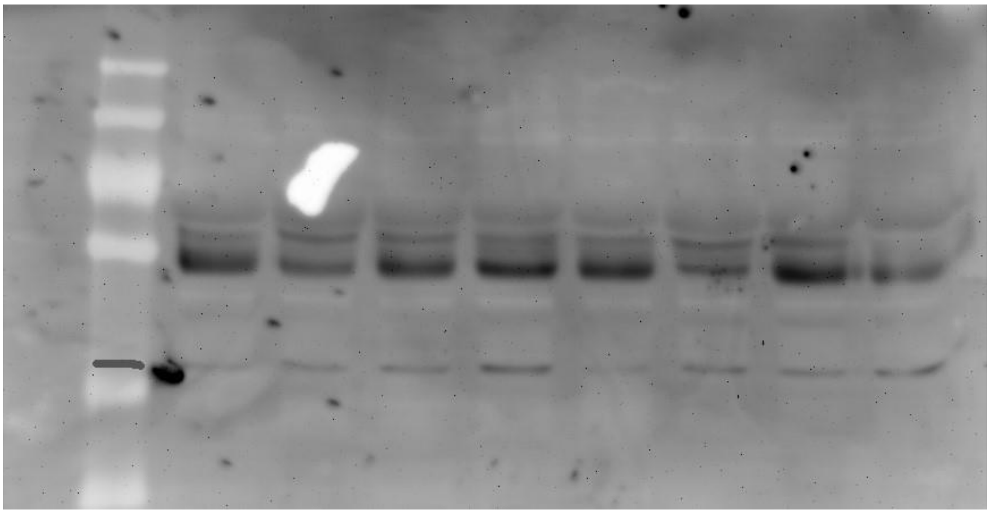

LPAR1 Fig. 2C

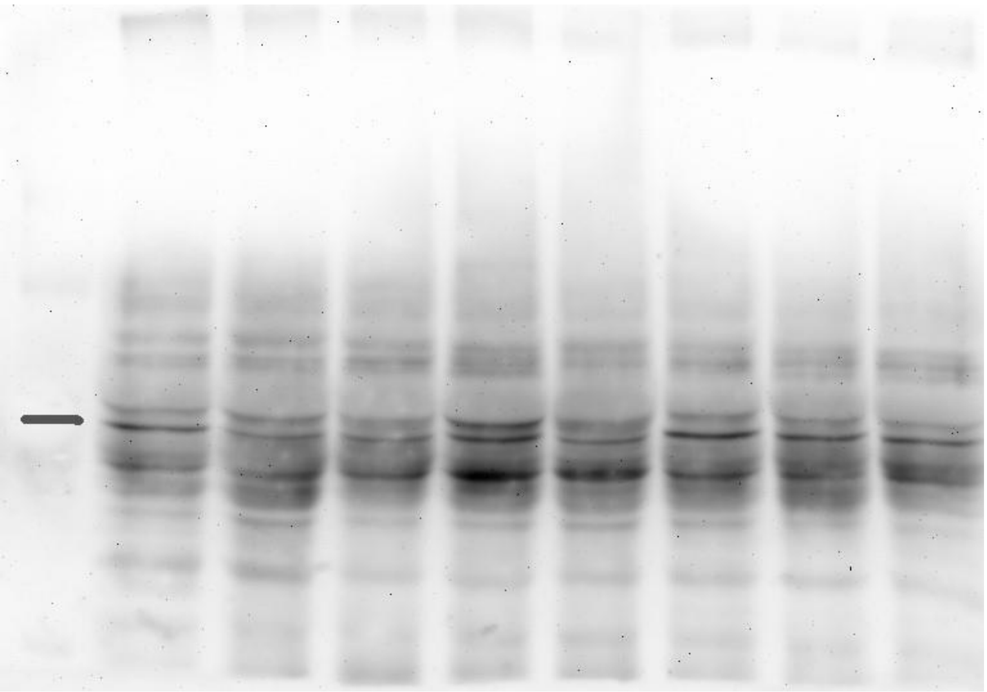

GAPDH Fig.2D

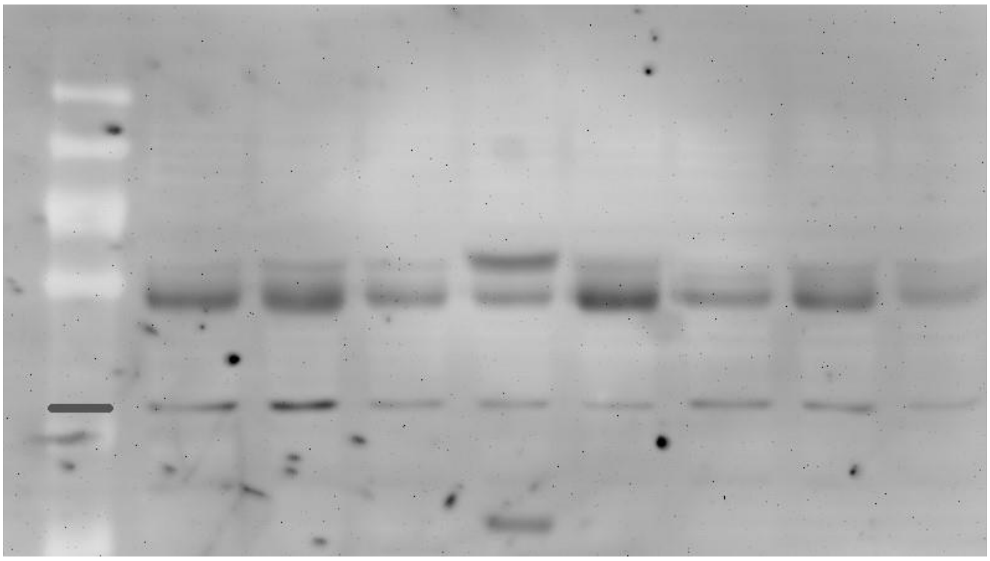

LPAR2 Fig. 2D

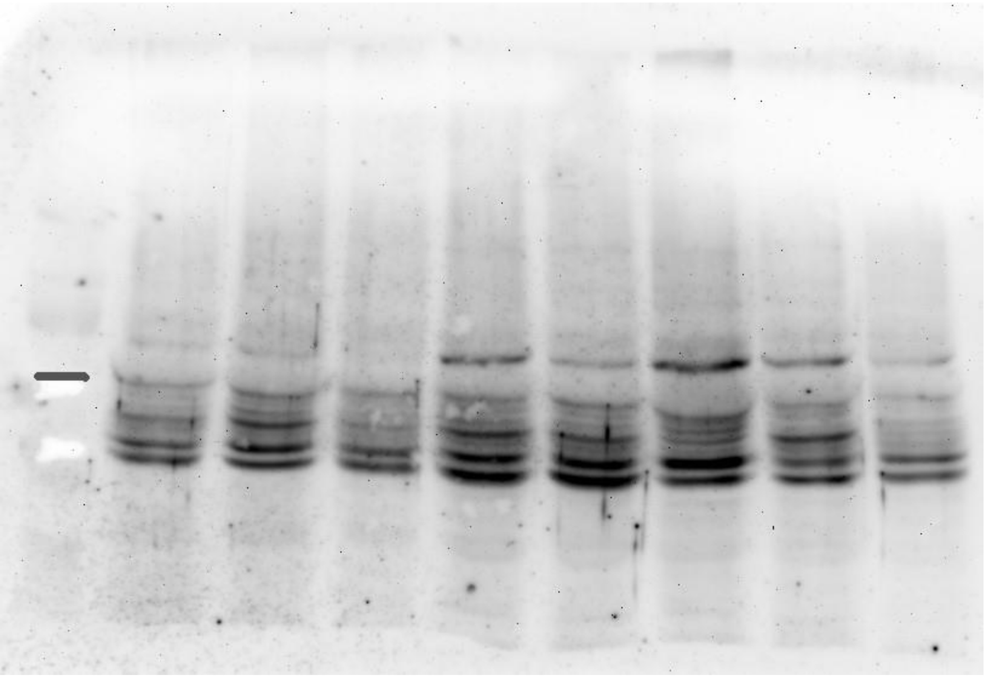

GAPDH Fig. 2G

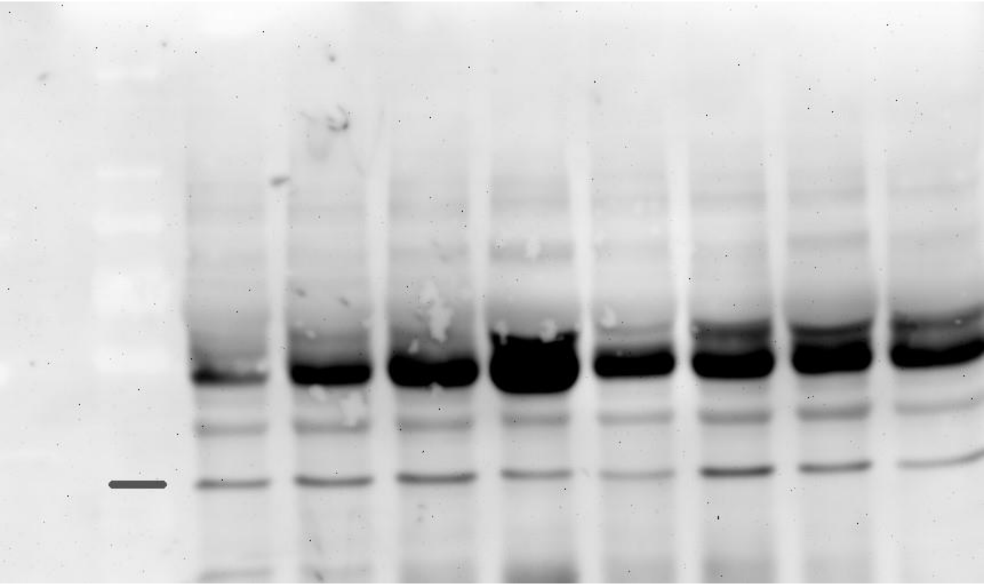

LPAR3 Fig.2G

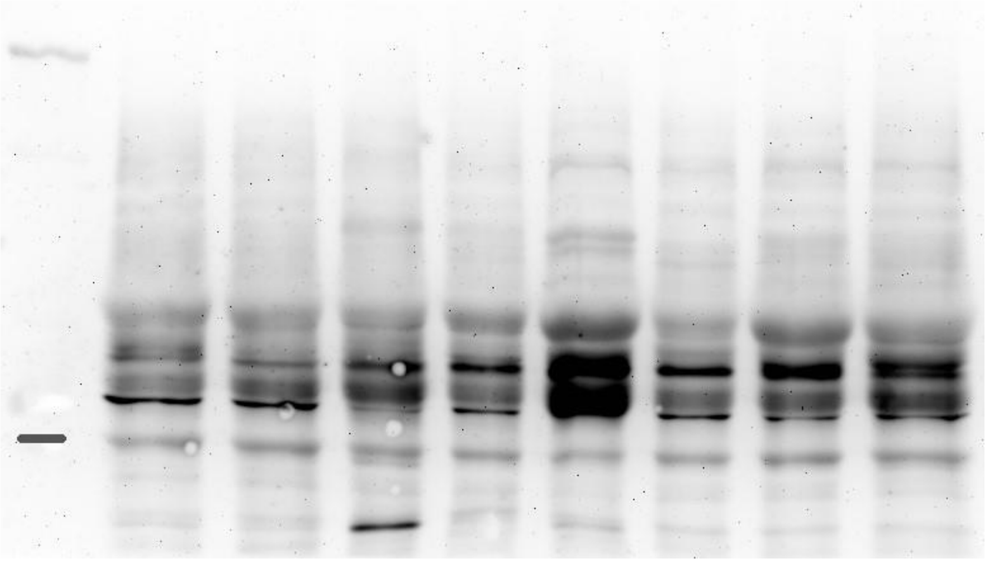

GAPDH Fig. 2H

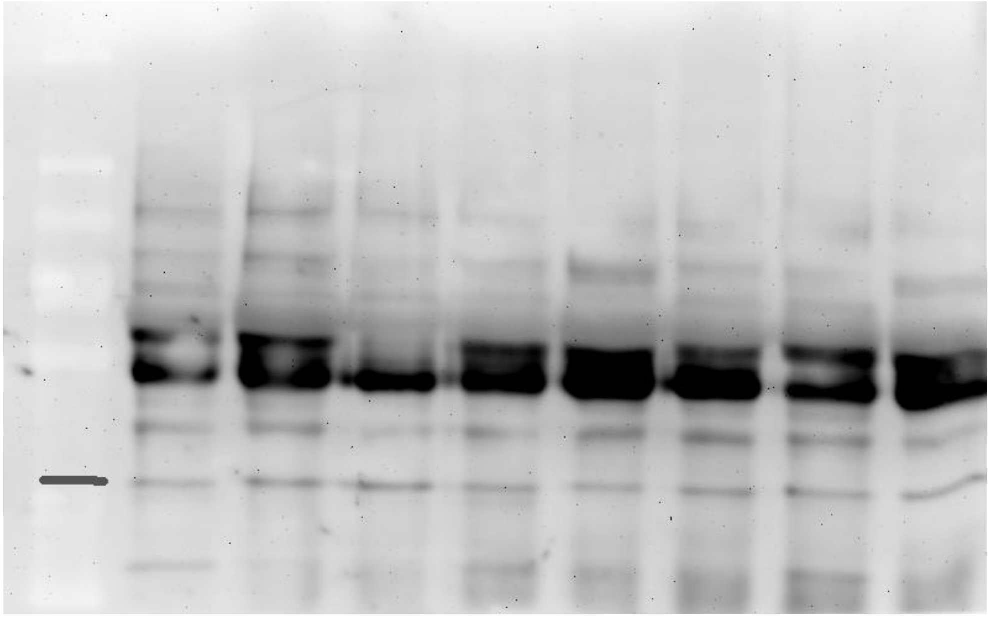

LPAR4 Fig.2H

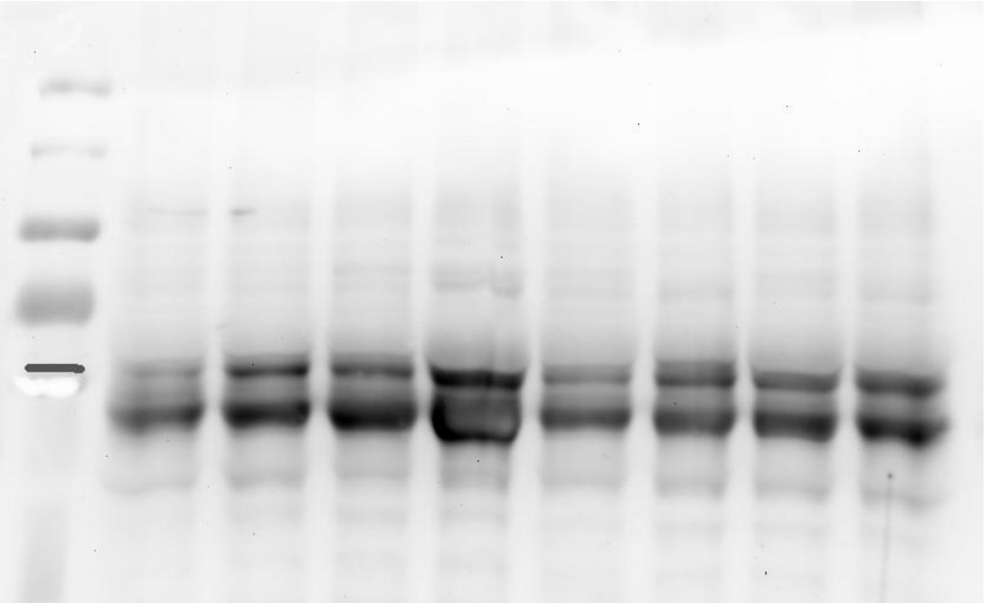

Supplement: Supplementary file 1 — Additional file 1. [file 12917_2020_2562_MOESM1_ESM.pdf]
