# Supplementary material for: Lysophosphatidic acid as a regulator of endometrial connective tissue growth factor and prostaglandin secretion during estrous cycle and endometrosis in the mare
Source: BMC Vet Res. 2020 Sep 17;16:343. doi: 10.1186/s12917-020-02562-6 (PMC7499873; doi:10.1186/s12917-020-02562-6)

Supplementary data 1

The effect of arachidonic acid (AA) at a dose 50 ng/ml on prostaglandin (PG)E_2 secretion in endometrial tissue at different stages of endometrosis. The responsiveness of tissues was tested by their incubation for 24h with AA as a positive control. The measurement of PGE_2 was performed using prostaglandin E_2 EIA kit (Cayman Chemical Company; #514010).

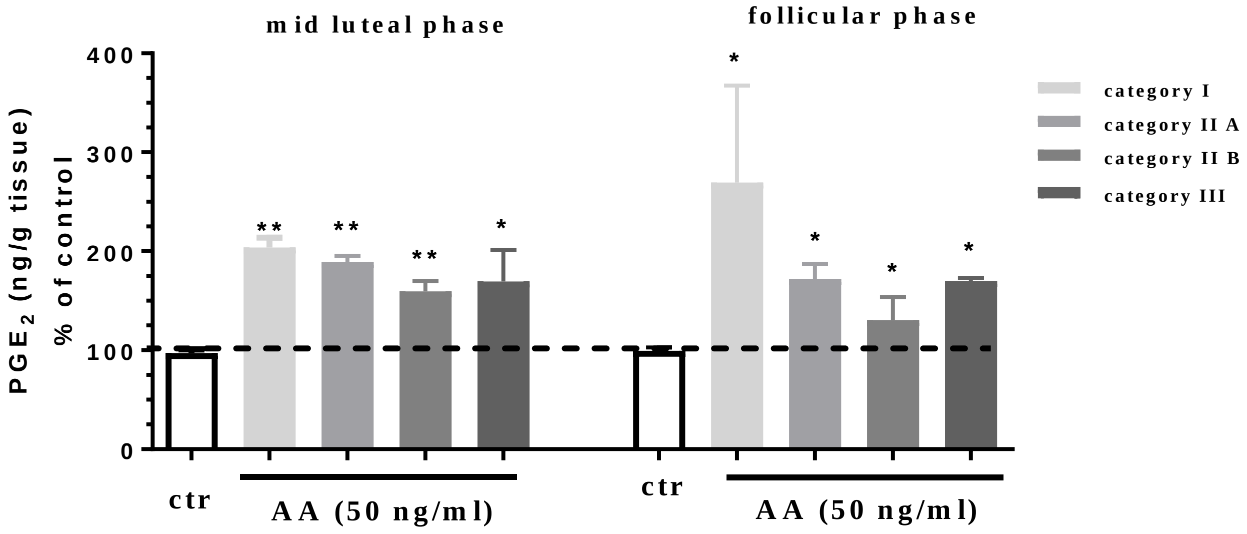

Supplement: Supplementary file 2 — Additional file 2. [file 12917_2020_2562_MOESM2_ESM.pdf]
